# Salt Priming Protects Photosynthetic Electron Transport against Low-Temperature-Induced Damage in Wheat

**DOI:** 10.3390/s20010062

**Published:** 2019-12-20

**Authors:** Hui Li, Huawei Li, Yanjie Lv, Yongjun Wang, Zongshuai Wang, Caiyun Xin, Shengqun Liu, Xiancan Zhu, Fengbin Song, Xiangnan Li

**Affiliations:** 1 Key Laboratory of Mollisols Agroecology, Northeast Institute of Geography and Agroecology, Chinese Academy of Science, Changchun 130102, China; helen199121@126.com (H.L.); lsq@iga.ac.cn (S.L.); songfb@iga.ac.cn (F.S.); 2 University of Chinese Academy of Science, Beijing 100049, China; 3 Crop Research Institute, Shandong Academy of Agricultural Sciences, Jinan 250100, China; lily984411@126.com (H.L.); wzshuai0109@163.com (Z.W.); 4 Institute of Agricultural Resources and Environment, Jilin Academy of Agriculture Sciences/State Engineering Laboratory of Maize, Changchun 130033, China; yjwang2004@126.com; 5 Rice Research Institute, Shandong Academy of Agricultural Science, 2 Sangyuan Road, Jinan 250100, China; yun.come2004@126.com; 6 College of Life Sciences, Anhui Normal University, Wuhu 241000, China; zhuxiancan@iga.ac.cn

**Keywords:** reactive oxygen species, cold stress, Chlorophyll a fluorescence, PS II reaction centers

## Abstract

Low temperature limits the photochemical efficiency of photosystems in wheat plants. To test the effect of salt priming on the photosynthetic electron transport in wheat under low temperature, the germinating seeds of a winter wheat cv. Jimai44 were primed with varying concentrations of NaCl solutions (0, 10, 30, and 50 mM NaCl, indicated by S0, S10, S30, and S50, respectively) for 6 d, and after 11 d of recovery, the seedlings were subsequently exposed to 24-h low-temperature stress (2 °C). Under low temperature, the S30 plants possessed the highest absorption flux per reaction center and higher density of reaction center per cross-section among the treatments. In addition, S30 plants had higher trapped energy flux for reducing Q_A_ and fraction of Q_A_-reducing reaction centers and non-Q_B_ reducing center than the non-primed plants under low temperature, indicating that S30 plants could maintain the energy balance of photosystems and a relatively higher maximum quantum efficiency of photosystem II under low temperature. In addition, the low temperature-induced MDA accumulation and cell death were alleviated by salt priming in S30 plants. It was suggested that salt priming with an optimal concentration of NaCl solution (30 mM) during seed germination enhanced the photochemical efficiency of photosystems in wheat seedlings, which could be a potential approach to improve cold tolerance in wheat at an early stage.

## 1. Introduction

Low-temperature stress limits wheat plant growth (*Triticum aestivum* L.), mainly due to the inhibition of photochemical efficiency in photosystems [1,2]. The photochemical process in photosystems is triggered when light is absorbed by the antenna, and excitation energy transferred from absorbed energy is either trapped at a reaction center for Q_A_-reducing and electron transport or dissipated as heat and emitting radiation-fluorescence [3]. A typical fluorescence transient, exhibiting the dark-adapted photosynthetic process by saturating light, is called OJIP (rapid fluorescence transient) curve [4,5]. This curve shows a sequence of phases from the initial (F_o_) to the maximal (F_M_) fluorescence value, which is labeled step O (all reaction centers open), J (~2 ms), I (~30 ms), P (equal to F_M_ when all reaction centers are closed) [3,4]. The shape of OJIP transient depends on the relative content of PS II, the redox state of the reaction centers and donors, cytochrome (Cybdf), PS I and the pool size of plastoquinone (PQ) and ferredoxin [3,4,5]. It has been well documented that low temperature causes an imbalance between the capacity for harvesting light energy and dissipating energy through metabolic activity, resulting in the damage of the thylakoid structure and distortion in the electron transport of photosynthetic reactions [6,7]. The leaf model of phenomenological energy fluxes showed that low temperature declines the trapped energy flux per cross-section (TR_o_/CS_M_) and electron transport flux per cross-section (ET_O_/CS_M_), hence leading to a significant decrease in quantum yield of chlorophyll fluorescence (F_V_/F_M_) [8,9,10]. Under low-temperature stress, the redundant excitation energy ultimately produces reactive oxygen species (ROS), and the oxidizing power potentially results in damage to the plant’s cell membrane [11,12].

The earlier moderate abiotic stress event might train plants to enhance the capability to survive from subsequent stresses, called ‘stress priming’, which is related to the modifications in physiological, proteomic and transcriptional levels [1,13]. Janda et al. (2016) demonstrated that salt priming with 25 mM NaCl improves salt tolerance via improving the osmotic potential and activating the antioxidant systems in wheat [14]. In addition, the salt treatment (125 mM NaCl) benefits for the photosynthetic carbon assimilation and growth in *Panicum turgidum* [15]. The seed priming with NaCl endows pepper plants with higher salt tolerance through inducing chlorophyll and proline accumulation [16]. In *Atriplex centralasiatica*, priming with 100 mM NaCl increases the thermotolerance by reducing PS II damage resulting in the increased maximum quantum efficiency of PS II and CO_2_ assimilation rate [17]. Likewise, in *Suaeda salsa L.*, priming with 200 mM NaCl increases the chilling tolerance by maintaining higher PS II efficiency, unsaturated fatty acid level and concentrations of some major membrane lipids, like DGDG [18,19]. However, the cross-tolerance induced by salt priming in wheat has received little attention.

To test the effect of salt priming on cold tolerance in wheat, the germinating seeds of a winter wheat cv. Jimai44 were primed with varying concentrations of NaCl solutions, and after recovery, the seedlings were subsequently exposed to low-temperature stress. The photochemical efficiency of photosystems in primed and non-primed wheat plants was studied under low-temperature stress. Our hypothesis was: (i) Salt priming in germinating seeds could improve the low-temperature tolerance in wheat seedlings, (ii) the effect of salt priming on photochemical efficiency of photosystems was dose-dependent.

## 2. Materials and Methods

### 2.1. Experimental Design and Materials

Seeds of winter wheat cv. Jimai44 were surface sterilized with 70% ethanol and 1% sodium hypochlorite solution and washed with distilled water. After sterilization, germinating seeds were primed with varying concentrations of NaCl solution, i.e., 10 mM (S10), 30 mM (S30), and 50 mM (S50). The seeds pre-soaked with just distilled water were set as the control (S0). The seed germination pouches were used for seed germinating and seedling establishment. Ten uniform seeds were put in each pouch, and 20 pouches were included in each treatment. The salt primed and the control seeds were incubated at 24 ± 0.5 °C for 6 d at dark in a growth chamber. Then, all seeds were transferred to new seed germination pouches containing Hoagland nutrient solution to recovery for 11 d at 24 °C/16 °C (day/night, 22,000 Lux/0 Lux) in the growth chamber. The relative humidity was 60%. After the recovery, wheat seedlings were exposed to 24-h low-temperature stress at 2 °C. Half of the wheat seedlings in S0 treatment were grown in normal temperature (NT, 24 °C/16 °C) as the normal temperature control.

### 2.2. Chlorophyll a Fluorescence

Just before the end of low-temperature treatment, the polyphasic rise in chlorophyll a fluorescence (OJIP) transient were measured using a portable fluorometer (Fluorpen FP100, Photon System Instruments, Drasov, Czech Republic). Plants were dark-adapted for 20 min by a detachable leaf-clip in the middle part of a leaf blade before measurements. Six leaves were selected in each treatment for the measurements. The measurements on each leaf were repeated three times and the average of three measurements for each leaf was calculated. The leaf samples were illuminated with continuous blue light (455 nm), and chlorophyll a fluorescence was induced by 1 s saturation pulses of 2400 μmol·m^−2^·s^−1^. In this study, a fast fluorescence rise from an initial fluorescence intensity F_O_ to a maximal intensity F_M_ was utilized: F_O_ was measured at 20 μs when all reaction centers are open, fluorescence intensity at 300 μs was denoted as F_300μs_ (K-step), fluorescence intensity F_J_ is at 2 ms (J-step), fluorescence intensity F_I_ is at 30 ms (I-step), and maximal fluorescence intensity F_M_ is reached after about 300 ms. In addition, the data obtained from raw fluorescence OJIP transients were treated according to the equations for calculations of JIP-test parameters, such as the maximal (subscript ‘O’) energy fluxes in the energy cascade for absorption (ABS), trapping (TR_O_), electron transport (ET_O_) and dissipation (DI_O_) [20].

Oxygen evolving complexes (OEC), an important fraction in PS II, were calculated by the adoption of the value of V_K_ and V_J_. The parameter R_J_ reflecting the relative changes of V_J_ offers an evaluation of the number of the reaction center (RC) with Q_B_-site filled by PS II inhibitor [20,21]. The amount of Q_A_ reaction centers and non-Q_A_ reducing centers were calculated as follows [20]:

Fraction of OEC centers = [1 − (V_K_/V_J_)]_treatment_/[1 − (V_K_/V_J_)]_control_, (1)

(2) $$\begin{array}{ll} R_{J} & =(V_{J(\mathrm{treatment})}-V_{J(\mathrm{control})})/(1-V_{J(\mathrm{control})}),\\ & =(\phi_{\mathrm{control}}-\phi_{\mathrm{treatment}})/\phi_{\mathrm{control}},\\ & =1-\phi_{\mathrm{treatment}}/\phi_{\mathrm{control}},\\ & =1-(\mathrm{ET}/\mathrm{TR}{)}_{\mathrm{treatment}}/({\mathrm{ET}}_{O}/{\mathrm{TR}}_{O}), \end{array}$$

Q_A_ reducing centers = (RC/RC_ref_)_.._(ABS/ABS_ref_),
= [(RC/CS)_treatment_/(RC/CS)_control_]·[(ABS/CS)_treatment_/(ABS/CS)_control_], (3)

The fraction of non-Q_A_ reducing centers = 100%-Q_A_ reducing centers, (4)

The PS II behavior was characterized based on the chlorophyll fluorescence parameters calculated by the following formulae in Table 1 [4,22,23].

### 2.3. Staining for Membrane Damage and Measurement of Malondialdehyde (MDA)

After low temperature, the last fully expanded leaves were harvested to study the membrane damage by Evan’ s blue staining [24]. Wheat leaves were immersed in Evan’ s blue solution (0.25 g of Evan’ s blue dissolved in 100 mL of 0.1 M CaCl_2_ solution at pH 5.6) and ensured to be stained. Then leaves were washed with 0.1 M CaCl_2_ solution at pH 5.6 thoroughly to remove all dye from the leave surface. The detection of Evan’s blue staining showed a ruptured and destabilized membrane. For MDA measurement, the fresh leaf (0.2 g) was homogenized in a 2 mL solution of 0.6% thiobarbituric acid and 5% trichloroacetic acid. The absorbance at 532 and 600 nm were measured spectrophotometrically according to Chen et al. [25].

### 2.4. Statistical Analysis

The statistical significance of differences was determined using one-way ANOVA followed by the post-hoc Duncan test to determine statistically homogenous groups with the software of SPSS 16.0 (SPSS Inc., Chicago, IL, US).

## 3. Results

### 3.1. Chl a Fluorescence Transient

**Figure 1:**
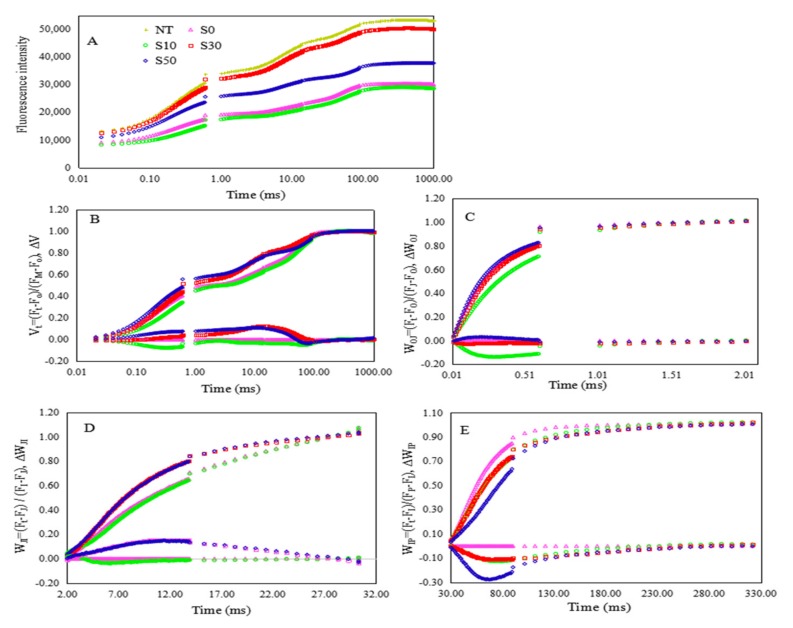
The chlorophyll a fluorescence transients of dark adapted leaves in wheat primed with varied concentrations of NaCl under low-temperature stress: (**A**) Raw fluorescence kinetics of leaves primed with varied concentrations of NaCl under low-temperature stress and the normal temperature control. (**B**) The fluorescence kinetics normalized by F_O_ and F_M_ as V_t_ = (F_t_ − F_O_)/(F_M_ − F_O_) (top), and ΔV_t_ = V_t (treatment)_ − V_t (control) (bottom)_. (**C**) The fluorescence kinetics normalized by F_O_ and F_J_ as W_OJ_ = (F_t_ − F_O_)/(F_J_ − F_O_) (top), and ΔW_OJ_ = W_OJ (treatment)_ − W_OJ (control)_ (bottom). (**D**) The fluorescence kinetics normalized by F_J_ and F_I_ as W_JI_ = (F_t_ − F_J_)/(F_I_ − F_J_) (top), and ΔW_JI_ = W_JI(treatment)_ − W_JI(control)_ (bottom). (**E**) The fluorescence kinetics normalized by F_I_ and F_P_ as W_IP_ = (F_t_ − F_I_)/(F_P_ − FI) (top), and Δ WIP = WIP (treatment) − WIP (control) (bottom). NT, non-treated leaves at room temperature; S0, the non-priming control; S10, priming with 10 mM NaCl; S30, priming with 30 mM NaCl; S50, priming with 50 mM NaCl.

A typical OJIP curve was observed in the treatment with non-salt priming under normal temperature. A significant difference was found in fluorescence transients (OJIP curves) between salt primed and non-primed plants under low temperature (Figure 1A). It was obvious that the fluorescence intensity of S30 was significantly higher than S0, S10, and S50, which was similar to the fluorescence intensity in NT. To study the mechanism of salt priming protected photosynthetic electron transport in wheat under low temperature, we further analyzed the normalized fluorescence kinetics and JIP-test parameters of S0, S10, S30, and S50 under low temperature.

The fluorescence transients at step O was not significantly affected by salt priming, while the major modifications caused by salt priming were found at step J, I and P. The normalized fluorescence transients between F_O_ (the step O, 20 μs) and F_M_ (peak) indicated that the fluorescence transients of S30 and S50 plants were less affected by low-temperature stress, compared with S0 and S10 plants (Figure 1B). In Figure 1C, the fluorescence transients were normalized between step O and step J in the linear time scale from 20 μs to 2 ms. The slightly positive value at K-band (at 300 μs) indicated a minor bypass of the OEC centers by non-water electrons in S50 plants, while no significant K-band was observed in S10 and S30 plants. It indicated that salt priming of S0 and S30 protected OEC from injury by low temperature. In addition, the W_OJ_ in S10 was significantly lower than those in other treatments, which indicated that S10 accelerated the accumulation of reduced Q_A_ in low temperature resulting in the inhibition of electron transferring to the second quinone electron acceptor Q_B_. Figure 1D,E showed the fluorescence transients double normalized between step J (2 ms) and P (the peak) in a linear time scale. The phase of J-I reflects a blockage of PS II electron up to PQ, and I-P phase showed PS I driven electron flow from PQH_2_ to the end electron acceptors on PS I acceptor side. In Figure 1D, a sharp increase in the J-I phase (W_JI_, from 2 to 30 ms) was observed in S30 and S50 plants. However, the W_IP_ in non-primed plants was significantly higher than that of salt primed plants (Figure 1E).

### 3.2. Electron Transport and Quantum Yield of PS II

**Figure 2:**
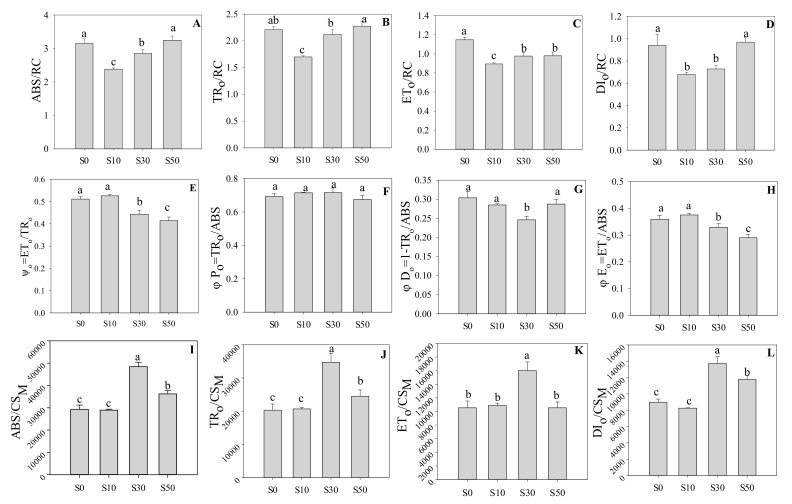
(**A**–**H**) The quantum efficiencies and energy fluxes quantifying the behavior of PS II in wheat leaves primed with varying concentrations of NaCl under low-temperature stress. (**I**–**L**) The phenomenological fluxes of excited cross-section in wheat primed with varying concentrations of NaCl under low-temperature stress. The explanation of JIP-test parameters is shown in Table 1. S0, the non-priming control; S10, priming with 10 mM NaCl; S30, priming with 30 mM NaCl; S50, priming with 50 mM NaCl. Different letters denote significant differences between treatments at *p* < 0.05.

The fluorescence transients were further analyzed to deduce the functional parameters quantifying the photosynthetic electron transport efficiency (Figure 2). S0 and S50 had the highest absorption flux per reaction center (ABS/RC), trapped energy flux per reaction center (TR_o_/RC), and dissipated energy flux per reaction center (DI_o_/RC), while S10 had the lowest values. The value of electron transport flux per reaction center (ET_o_/RC) was the highest in S0, compared with primed plants. In addition, no significant difference was found in φP_o_ (the maximum quantum yield for PS II primary photochemistry). S0 and S10 had significantly higher ψ_o_ (the probability that a trapped exciton moves an electron into the electron transport chain beyond Q_A_) and φE_o_ (the quantum yield for PS II electron transport), compared with S30 and S50. The parameter of φD_o_ (the quantum yield for dissipation) in S30 was significantly lower than other treatments. Furthermore, the phenomenological fluxes of excited cross-section, including ABS/CS_M_ (absorption energy flux per cross-section), TR_o_/CS_M_ (trapped energy flux), ET_o_/CS_M_ (electron transport flux) and DI_o_/CS_M_ (dissipated energy flux), in S30 were significantly higher than other treatments (Figure 2I–L). The results indicated that S30 protected photosynthetic electron transport against low temperature through increasing active reaction centers per cross-section.

### 3.3. Technical Fluorescence Parameters in PS II

**Figure 3:**
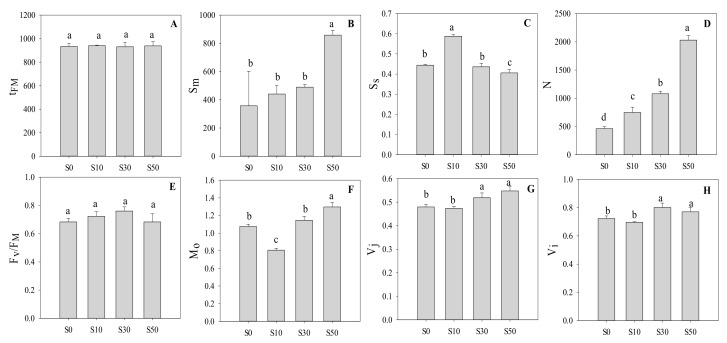
The technical fluorescence parameters based on different processes of chlorophyll a fluorescence transients in wheat leaves primed with varying concentrations of NaCl under low-temperature stress. (**A**) Time to reach the maximal fluorescence intensity; (**B**) Normalized total complementary area above the O-J-I-P transient; (**C**) Normalized total complementary area corresponding only to the O-J phase; (**D**) Frequency of Q_A_ reduction; (**E**) The maximal quantum yield of PS II; (F) Approximated initial slope of fluorescence transient normalized on the maximal variable fluorescence; (**G**) Relative variable fluorescence at J-step; (**H**) Relative variable fluorescence at I-step. S0, the non-priming control; S10, priming with 10 mM NaCl; S30, priming with 30 mM NaCl; S50, priming with 50 mM NaCl. Different letters denote significant differences between treatments at *p* < 0.05.

The time for achieving the balance between PS I and PS II (t_FM_) was similar in these four treatments (Figure 3). The S_m_ and N were highest in S50, followed by S30 and S10, while the lowest values were found in S0, which reflects multiple-turnover Q_A_ reduction events and frequency of Q_A_ reduction from O to t_FM_, respectively. However, the S_S_ (single-turnover Q_A_ reduction events) was highest in S10, followed by S0 and S30, and the lowest in S50. The highest M_O_ was found in S50, followed by S0 and S30, while that of S10 was the lowest. A similar trend was found in both increment of variable fluorescence in the step-J (V_j_) and step-I (V_i_), where that in S30 and S50 was significantly higher the one in S0 and S10 plants.

### 3.4. Reaction Centers in PS II

**Figure 4:**
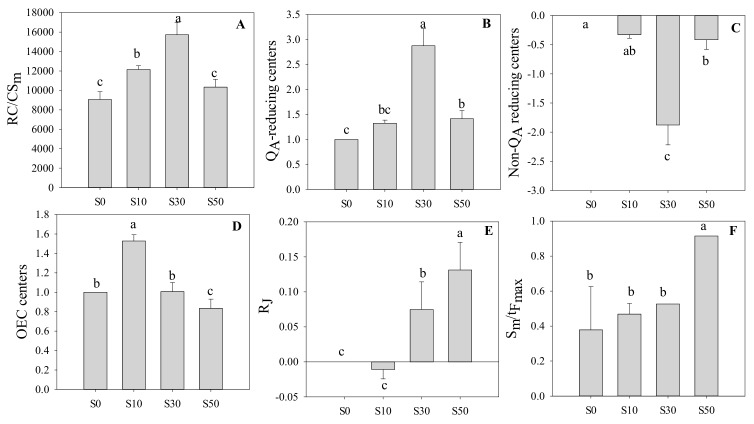
The density of RCs in wheat leaves primed with varying concentrations of NaCl under low-temperature stress. (**A**) Q_A_-reducing RCs per cross section; (**B**) The fraction of Q_A_-reducing RCs; (**C**) The fraction of non-Q_A_ reducing RCs; (**D**) The fraction of OEC; (**E**) The number of PS II RCs with Q_B_-site filled by PS II inhibitor; (**F**) Average fraction of open RCs of PS II. S0, the non-priming control; S10, priming with 10 mM NaCl; S30, priming with 30 mM NaCl; S50, priming with 50 mM NaCl. Different letters denote significant differences between treatments at *p* < 0.05.

In Figure 4, The highest RC/CS_m_ was found in S30 plants, followed by S10, S50, and S0, but there was no significant difference between S0 and S50, which indicated that S30 had the highest number of active reaction centers per cross-section. In Figure 4B, salt priming significantly increased Q_A_ reducing centers in wheat under low temperature, compared with S0, and S30 had the highest Q_A_ reducing centers. In contrast, salt priming significantly decreased non-Q_A_ reducing centers compared with S0, and S30 had the lowest non-Q_A_ reducing centers. Furthermore, the highest OEC centers (the fraction of oxygen-evolving complexes) was found in S30, followed by S0 and S30, while that of S50 was the lowest. As shown in Figure 4E, compared with S0, the S30 and S50 significantly increased R_J_. The R_J_ was lower significantly in S10, in relation to S0. R_J_ reflects the number of PS II RCs with Q_B_-site filled by PS II inhibitor, the results indicated that S50 decreased the probability that a trapped exciton moves an electron into the electron transport chain beyond Q_B_. In addition, S50 had the highest ratio of S_m_/t_Fmax_, while there was no significant difference among the S0, S10 and S30 plants, which showed that S50 caused a postpone closure of RC in PSII due to the enhancement of the average fraction of open RC in the time interval from 0 to t_Fmax_.

### 3.5. Performance Index and Driving Force in PS II

**Figure 5:**
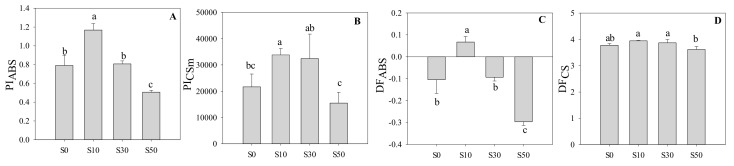
(**A**) Performance index for energy conservation from photons absorbed by PS II to the reduction of intersystem electron acceptors, (**B**) performance index based on cross section, (**C**) driving force based on absorption of light energy, and (**D**) driving force based on cross section in wheat leaves primed with varying concentrations of NaCl under low-temperature stress. S0, the non-priming control; S10, priming with 10 mM NaCl; S30, priming with 30 mM NaCl; S50, priming with 50 mM NaCl. Different letters denote significant differences between treatments at *p* < 0.05.

In Figure 5, S10 had the highest PI_ABS_, followed by S0 and S30, while S50 had the lowest PI_ABS_. A similar trend was found in PI_CSm_, where the S10 and S30 plants had the highest PI_CSm_, while S50 plants had the lowest values. In addition, the DF_ABS_ in S10 plants was significantly higher than others, while the lowest value was found in S50 plants. The DF_CS_ in S30 and S10 plants were the highest among these treatments.

### 3.6. Membrane Damage and Malondialdehyde of Leave

**Figure 6:**
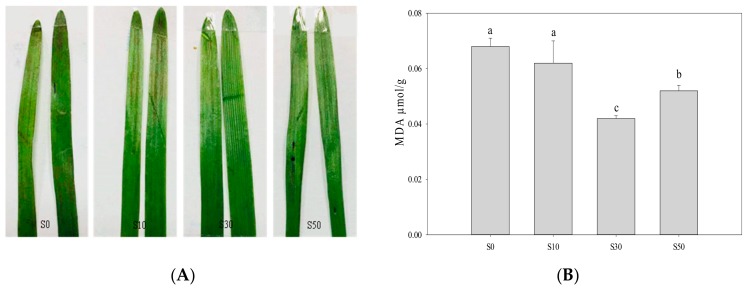
(**A**) Detection of membrane damage by Evan’s blue staining in wheat leaves primed with varying concentrations of NaCl under low-temperature stress. (**B**) Malondialdehyde (MDA) concentration in wheat primed with varying concentrations of NaCl under low-temperature stress. S0, the non-priming control; S10, priming with 10 mM NaCl; S30, priming with 30 mM NaCl; S50, priming with 50 mM NaCl. Different letters denote significant differences between treatments at *p* < 0.05.

In Figure 6A, it was observed that the membrane damage indicated by Evan’s blue staining was lower in S30 plants under low temperature, compared with others. The MDA concentration was the highest in S0 and S10, followed by S50, and the MDA concentration was the lowest in S30 (Figure 6B).

## 4. Discussion

Low-temperature stress affects membrane stability resulting in overproduction of ROS, which inhibits photosynthetic electron transport [1,18]. It is well known that the fast chlorophyll a fluorescence induction curve has been wildly used to study the response of photosynthetic electron transport to low-temperature stress [2]. In the study, the chlorophyll fluorescence transients in salt primed wheat plants were less affected by low temperature, in relation to non-primed plants, indicating that salt priming alleviated the low-temperature-induced damage to photosystems. The catalytic reaction of oxygen evolution in OEC centers occurs as a result of H_2_O oxidation, which functions as a terminal electron donor of the electron transfer chain in photosynthesis [26,27]. In this study, a slightly negative K-band was found in S10 plants, indicating that salt priming with a relatively low concentration of NaCl aggravated the damage of OEC and limited the electron transfer chain. However, S30 plants had larger amounts of active OEC centers than S0 plants. This demonstrated that salt priming with 30 mM NaCl could protect OEC from damage induced by low temperature, which contributed to the higher electron transport efficiency in salt primed plants. In Figure 4, the results showed that S50 increased the fraction of open RC. However, S50 plants had the lowest OEC centers, while significantly higher DI_o_/RC. These indicated that the energy flux absorbed by the reaction center was mostly dissipated as heat in relatively high NaCl priming, which might result in lower ATP production.

The PI_ABS_ is one of the most sensitive parameters to abiotic stress, which is related to photochemical and non-photochemical properties and the density of active reactive centers per chlorophyll absorption [4]. The PI_ABS_ is regulated by three components, including RC/ABS, TR_o_/ABS, and ET_o_/TR_o_, suggesting the change of PI_ABS_ is attributed to antenna size, the maximum quantum yield of primary photochemistry and the probability of electron obtained by Q_A_ moving further into electron transport chain [4,20]. RC/ABS is the reciprocal value of ABS/RC, which reflects the antenna size to regulate the stability of the reaction centers and their connection to the antenna and light-harvesting complexes. In the present study, no significant difference was found in TRo/ABS. However, ET_o_/TR_o_ in S30 was significantly lower than non-primed plants, and ABS/RC was significantly decreased by S30, compared with S0. This indicated that the main factor influencing PI_ABS_ was the change of ability to absorb light energy for the reaction center. S30 increased the absorb energy through increasing active reaction centers per cross-section instead of absorption flux per RC. It was further proved by the results that S30 plants had the highest PI_CSm_ to produce higher energy attending the reduction of intersystem electron acceptors in wheat under low temperature. However, higher trapped energy for reducing Q_A_ (TR_o_/RC) in S30 than that in S10 indicated that the electron was blocked in the process of transport from Q_A_ to the second quinone electron acceptor Q_B_ in S10 plants. The results were in agreement with the observation of W_OJ_.

The features of the emitted fluorescence are determined by the transferring of excitation energy. Current models of the chlorophyll fluorescence transients showed that step J coincides with the maximum concentrations of Q_A_^−^Q_B_ and Q_A_^−^Q_B_^−^, equivalent to the process of electron transport from Q_A_ to Q_B_. The O-J phase is largely driven by primary photochemistry involving the closure of PS II reaction centers due to the single-turnover Q_A_ reduction [27]. The step I is corresponds to the concentration of Q_A_^−^Q_B_^2−^, whereas, the step P corresponds to the maximum concentrations of both Q_A_^−^Q_B_^2−^ and PQH_2_, reflecting the process of electron transport from Q_B_ to PQ. At the J-P phase, multiple-turnover Q_A_ reduction is conducted to reflect the biochemical process [5]. It has been well known that low-temperature stress limits the photochemical utilization capacity of light energy [2]. Here, the findings showed a dose-dependent increase of V_j_, showing that salt priming in S30 mainly influenced the redox reaction after Q_A_ due to the interruption of the electron flow beyond Q_A_ in wheat under low temperature. Moreover, the changes in the OJ phase showed no significant difference between salt primed and non-primed plants. Thus, the salt priming (S30) significantly affected the multiple-turnover action for the reduction of Q_A_^−^, Q_B_^−^, and Q_B_^2−^, instead of the single-turnover Q_A_ reduction.

The frequency of Q_A_ reduction (N) in S30 plants was significantly higher than S0 plants, indicating that S30 plants had more frequency of Q_A_ reduction than non-primed plants. The J-I phase corresponds to the reduction of the secondary electron acceptor Q_B_, PQ, Cytb6f and PC [28]. The reaction centers, which are not capable of transferring electrons from Q_A_ to Q_B_, are referred to as Q_A_ reducing centers or non-Q_B_ reducing centers [29]. In the present study, W_JI_ was sharply increased in S30 plants than others, and Q_A_ reducing centers in S30 plants was significantly higher than others. This documented that the amount of electron in S30 plants was reduced as Q_A_^−^ or Q_A_^2−^ in Q_A_ reducing centers, compared with non-primed plants. The I-P phase reflects PS I drove the electron flow from PQH_2_ to the end electron acceptors of PS I acceptor side [20]. Here, it was found that S30 plants possessed significantly lower W_IP_, in relation to non-primed plants. This demonstrated that salt priming alleviated the damage to PS I electron acceptor under low temperature, hence promoting the electron transport from PQH_2_ to PS I acceptor side in S30 plants. Thus, the energy balance between PS I and PS II reached earlier in S30 plants under low temperature.

One of the major sources of ROS production is the photosynthesis process [30]. The ROS are produced in low-temperature stress [19]. The overproduction of ROS in chloroplasts results in photodamage, which reduces the photosynthetic electron transport efficiency [30]. Another effect of ROS is lipid peroxidation via oxidation of unsaturated fatty acids leading to membrane damage, and the MDA level has been used as an indicator for lipid peroxidation [12]. There is evidence that cold priming can activate the sub-cellular antioxidant systems and alleviates oxidative burst in photosynthetic apparatus to improve the tolerance to subsequent cold stress in wheat [2]. It is therefore tempting to speculate that salt priming could be a potential approach like cold priming to improve cold tolerance in wheat. The results in the present study were consistent with the results in *Suaeda salsa* [18]. It was reported that the membrane damage indicated by Evan’s blue staining and MDA concentration were lower in S30 plants under low temperature, compared with non-primed plants. Thus, salt priming of 30 mM NaCl alleviated the MDA accumulation induced oxidative damage to the photosynthetic apparatus, which maintained the relatively higher efficiency of photosynthetic electron transport and increased cold tolerance for wheat under low-temperature stress.

## 5. Conclusions

Under low temperature, the S30 plants had the highest absorption flux per reaction center and higher density of reaction center per cross-section among the treatments. Moreover, S30 plants had higher trapped energy flux for reducing Q_A_ and fraction of Q_A_^−^ reducing reaction centers and non-Q_B_ reducing center than the non-primed plants under low temperature, indicating that S30 plants could maintain the energy balance of photosystems and a relatively higher maximum quantum efficiency of PS II under low temperature. In addition, the low-temperature-induced MDA accumulation and cell death were alleviated by salt priming in S30 plants. It was concluded that salt priming with an optimal concentration of NaCl solution (30 mM) during seed germination enhanced the photochemical efficiency of photosystems in wheat seedlings, which could be a potential approach to improve cold tolerance in wheat at an early stage. In addition, these results revealed many differences in the function of PS II of wheat primed with salt under low temperature. However, whether these differences can be present during the priming period at ambient temperature and whether they can also manifest over a longer light period, for instance at steady-state level of photosynthesis, still need further investigations.

## Figures and Tables

**Table 1:** Formulae and explanation in the technical data of OJIP curves (rapid fluorescence transient) and the selected JIP-test parameters used in the study.

**Technical Fluorescence Parameters.**	**Illustrations**
F_o_ ≌ F_20μs_	Minimal fluorescence, when all PSII RCs are open
F_K_ ≡ F_300μs_	Fluorescence intensity at the K-step (300 μs) of OJIP
F_J_ = F_2ms_	Fluorescence intensity at the J-step (2 ms) of OJIP
F_I_ = F_30ms_	Fluorescence intensity at the I-step (30 ms) of OJIP
F_p_(= F_M_)	Maximal recorded fluorescence intensity, at the peak P of OJIP
t_FM_	Time (in ms) to reach the maximal fluorescence intensity F_M_
Area	Total complimentary area between the fluorescence induction curve and F = F_M_
V_j_ = (F_J_ − F_o_)/(F_M_ − F_o_)	Relative variable fluorescence at the J-step
M_o_ = 4·(F_270μs_ − F_o_)/(F_M_ − F_o_)	Approximated initial slope (in m·s−^1^) of the fluorescence transient normalized on the maximal variable fluorescence F_V_
S_m_ = Area/(F_M_ − F_o_)	Normalized total complementary area above the O-J-I-P transient, reflecting multiple-turnover Q_A_ reduction events
S_S_ = V_J_/M_o_	Normalized total complementary area corresponding only to the O-J phase, reflecting single-turnover Q_A_ reduction events
N = S_m_/S_S_ = S_m_·M_o_·(1 − V_J_)	Frequency of Q_A_ reduction from t = 0 to t = t F_M_
**Quantum Efficiencies or Flux Ratios**	**Illustrations**
ψ_o_ = PS I_o_ = ET_o_/TR_o_ = (1 − V_J_)	Probability that a trapped exciton moves an electron into the electron transport chain beyond Q_A_ at t = 0
φP_o_ = TR_o_/ABS = 1 − F_o_/F_M_ = F_V_/F_M_	Maximum quantum yield for PS II primary photochemistry at t = 0
φD_o_ = 1 − φP_o_ = F_o_/F_M_	Quantum yield for dissipation at t = 0
φE_o_ = ET_o_/ABS =(1 − F_o_/F_M_)·(1 − V_J_)	Quantum yield for PS II electron transport at t = 0 (ET)
**Specific Energy Fluxes (per Q_A_-reducing PS II reaction center-RC)**	**Illustrations**
ABS/RC = M_o_·(1/V_J_)·(1/φP_o_)	Absorption flux per reaction center (RC)
TR_o_/RC = M_o_·(1/V_J_)	Trapped energy flux per RC at t = 0
ET_o_/RC = M_o_·(1/V_J_)·ψ_o_	Electron transport flux per RC at t = 0
DI_o_/RC = ABS/RC − TR_o_/RC	Dissipated energy flux per RC at t = 0
**Phenomenological Energy Fluxes (per excited cross section-CS)**	**Illustrations**
ABS/CS_M_≈ F_M_	Absorption flux per cross section (CS) at t = t_FM_
TR_o_/CS_M_ = φP_o_·(ABS/CS_M_)	Trapped energy flux per CS at t = t_FM_
ET_o_/CS_M_ = φE_o_·(ABS/CS_M_)	Electron transport flux per CS at t = t_FM_
DI_o_/CS_M_ = ABS/CS_M_ − TR_o_/CS_M_	Dissipated energy flux per CS at t = t_FM_
**Density of RCs**	**Illustrations**
RC/CS_M_ = φP_o_·(V_J_/M_o_)·(ABS/CS_M_)	Q_A_- reducing RCs per CS, reflecting density of RCs at t = t_FM_
QA-reducing centers = (RC/RC_reference_)·(ABS/ABS_reference_) = ((RC/CS)_treatment_/(RC/CS)_control_) ·((ABS/CS) _treatment_/(ABS/CS)_control_)	The fraction of Q_A_-reducing reaction centers
Non-Q_A_ reducing centers = 1-Q_A_-reducing centers	The fraction of non-Q_A_ reducing reaction centers
OEC centers = (1 − (V_K_/V_J_) _treatment_)/(1 − (V_K_/V_J_) _control_)	The fraction of oxygen-evolving complexes (OEC)
R_J_ = (ψE_o (control)_ − ψE_o (treatment)_)/ψE_o (control)_ = (V_J (treatment)_ − V_J (control)_)/(1 − V_J (control)_)	Number of PS II RCs with Q_B_-site filled by PS II inhibitor
S_m_/t_Fmax_ = [RC_open_/(RC_close_+RC_open_)]av = [Q_A_/Q_A(total)_]av	Average fraction of open RCs of PS II in the time span between 0 and t_FM_
**Performance Index and Driving Force**	**Illustrations**
PI_ABS_ = (RC/ABS)·[φP_o_/(1 − φP_o_)]·[ψ_o_/(1 − ψ_o_)]	Performance index (potential) for energy conservation from photons absorbed by PS II to the reduction of intersystem electron acceptors
PICS_m_ = (RC/CS_M_)·[φP_o_/(1 − φP_o_)]·[ψ_o_/(1 − ψ_o_)]	Performance index based on cross section at t = t_FM_
DF_ABS_ = log(PI_ABS_)	Driving force based on absorption of light energy
DF_CS_ = log(PI_CS_)	Driving force based on cross section
